# The China Image Set (CIS): A New Set of 551 Colored Photos With Chinese Norms for 12 Psycholinguistic Variables

**DOI:** 10.3389/fpsyg.2019.02631

**Published:** 2019-12-04

**Authors:** Long Ni, Ye Liu, Wenyuan Yu, Xiaolan Fu

**Affiliations:** ^1^State Key Laboratory of Brain and Cognitive Science, Institute of Psychology, Chinese Academy of Sciences, Beijing, China; ^2^Department of Psychology, University of the Chinese Academy of Sciences, Beijing, China

**Keywords:** image set, shape diagnosticity, manipulation experience, Chinese norms, naming latency

## Abstract

Normative image sets are widely used in memory, perception, and language studies. Following the pioneering work of Snodgrass and Vanderwart (1980), a number of normalized image sets with various language norms have been created. However, original image sets that are carefully selected to accommodate Chinese culture and language are still in short supply. In the present study, we provided the China Image Set (CIS), a new set of photo stimuli with Chinese norms. The CIS consists of 551 high-quality colored photo stimuli that cover 21 categories and are normalized on 12 different variables, including name agreement, category agreement, familiarity, visual complexity, object manipulability, manipulation experience, color diagnosticity, shape diagnosticity, image variability, age of acquisition, image agreement, and within-category typicality. Of the 12 variables, shape diagnosticity and manipulation experience with the object depicted in a stimulus are the two newly introduced and normalized variables. Multiple regression analysis reveals that name agreement, age of acquisition, image agreement, shape diagnosticity, and image variability are the most robust determinants of picture naming latency. Our normative dataset of the high-quality photo stimuli offers an ecologically more valid tool to study object recognition and language processing within Chinese culture than has previously been available.

## 1. Introduction

Image materials have been widely used in the studies of object identification (e.g., Tipper, 1985; Humphreys et al., 1988; Martin et al., 1996; Stanfield and Zwaan, 2001), memory (e.g., Snodgrass and Corwin, 1988; Kelley et al., 1998; Alvarez and Cavanagh, 2004; Patterson et al., 2007), and language processing (e.g., Damasio et al., 1996, 2004; Vandenberghe et al., 1996; Bozeat et al., 2000) in both healthy subjects and patients. Snodgrass and Vanderwart (1980) did the pioneering work of providing the first standardized set of pictures. The original set of 260 black and white line drawings was normalized on five variables: name agreement, image agreement, familiarity, visual complexity and image variability.

### 1.1. Pioneering Work of Snodgrass and Vanderwart (1980)

Name agreement indicates the degree to which people agree on the dominant name of a specific object. Numerous studies have indicated that pictures with high name agreement have shorter naming latency (e.g., Snodgrass and Yuditsky, 1996; Barry et al., 1997; Ellis and Morrison, 1998; Cuetos et al., 1999; Bonin et al., 2002; Pind and Tryggvadóttir, 2002; Alario et al., 2004; Severens et al., 2005; Weekes et al., 2007; Liu et al., 2011) and shorter object comprehension latency (e.g., Bonin et al., 2013) than those with low name agreement. More familiar concepts were named faster, retrieved more readily from their corresponding category, and also categorized faster than unfamiliar concepts (e.g., Cuetos et al., 1999; Pind and Tryggvadóttir, 2002; Weekes et al., 2007; Liu et al., 2011). The visual complexity of an object picture is reflected in its amount of visual texture or the intricacy of its lines. There is evidence showing that observers take a longer time to name and categorize visually more complex pictures (e.g., Ellis and Morrison, 1998; Alario et al., 2004). Image agreement, defined as the extent to which an object photo resembles the observer's mental image, and image variability, a measure of the extent to which an object name evokes few or many different images for a particular object, were also found to influence object recognition and memory processing. Specifically, pictures with high image agreement and image variability were named and categorized faster than pictures with low image agreement and image variability (e.g., Barry et al., 1997; Ellis and Morrison, 1998; Bonin et al., 2002; Alario et al., 2004). Snodgrass and Vanderwart (1980) also obtained age of acquisition (AoA) norms for 89 pictures from Carroll and White (1973) and Snodgrass and Yuditsky (1996) provided AoA ratings for 250 of the original 260 pictures. AoA is one of the most robust determinants of naming latency reported in the literature (Ellis and Morrison, 1998; Cuetos et al., 1999; Bonin et al., 2002; Alario et al., 2004; Severens et al., 2005; Weekes et al., 2007; Liu et al., 2011), and has been found to modulate the effect of word frequency on naming latency (Carroll and White, 1973; Morrison et al., 1992; Pind and Tryggvadóttir, 2002).

### 1.2. Following Work

Ever since the pioneering work of Snodgrass and Vanderwart (1980), those who followed either re-normalized the same set of pictures for different languages or proposed new sets of images with the standards set by Snodgrass and Vanderwart (1980). For the former group of studies, norms for the same set of black and white line drawings have been provided for Russian (Tsaparina et al., 2011), Turkish (Raman et al., 2014), Dutch (Martein, 1995), Japanese (Nishimoto et al., 2005), French (Alario and Ferrand, 1999), Spanish (Cuetos et al., 1999), IceIandic (Pind and Tryggvadóttir, 2002), Greek (Dimitropoulou et al., 2009), Italian (Nisi et al., 2000), Chinese (Shu and Cheng, 1989; Zhang and Yang, 2003; Weekes et al., 2007; Liu et al., 2011), and Persian (Bakhtiar et al., 2013). For the latter group of studies, researchers proposed various new sets of colored photos (e.g., Adlington et al., 2009; Brodeur et al., 2010, 2014; Zhou and Chen, 2017) that were normalized on a wide range of variables.

The latter proposed new image sets enrich the work of Snodgrass and Vanderwart (1980) in three important dimensions. First, most of the new datasets consist of high-quality colored photos that have higher ecological validity than the black and white line drawings in Snodgrass and Vanderwart (1980) (see Viggiano et al., 2004; Adlington et al., 2009; Brodeur et al., 2010, 2014; Zhou and Chen, 2017). Evidence has indicated that adding textures and colors to the line drawings shortened the picture naming latencies and facilitated object recognition (Ostergaard and Davidoff, 1985; Davidoff and Ostergaard, 1988; Price and Humphreys, 1989; Brodie et al., 1991).

Second, besides the five variables that were normalized in Snodgrass and Vanderwart (1980), the later proposed normative datasets have introduced new variables, including object manipulability, color diagnosticity, within-category typicality, and category agreement. Specifically, Magnié et al. (2003) first introduced object manipulability, defined as the degree to which an object can be manipulated by our hands (see also Brodeur et al., 2010; Salmon et al., 2010; Guérard et al., 2015). A number of behavioral and neurophysiological studies have indicated that action knowledge regarding how to manipulate an object is heavily involved in object recognition and likely to be integrated into the semantic representation of object concepts (e.g., Buxbaum and Saffran, 2002; Helbig et al., 2006; Vainio et al., 2008; Bub et al., 2013; Ni et al., 2014, 2019). Object manipulability also has been found to be a significant predictor of naming latency. Objects that are easy to grasp were named more rapidly (Guérard et al., 2015; Lorenzoni et al., 2018). Rossion and Pourtois (2004) introduced the variable of color diagnosticity, defined as the degree of association between an object and a specific color (see also Adlington et al., 2009). Color information has been shown to facilitate the recognition of objects with highly diagnostic colors, suggesting that color information is an intrinsic component of object representation (Ostergaard and Davidoff, 1985; Wurm et al., 1993; Tanaka and Presnell, 1999; Naor-Raz et al., 2003; Rossion and Pourtois, 2004; Therriault et al., 2009; Bramão et al., 2011). Dell'acqua et al. (2000) introduced a new variable of within-category typicality, which is defined as the degree to which a concept is a representative exemplar of its category (see also Lotto et al., 2001; Moreno-Martínez et al., 2011). Typical objects within a category were named and categorized faster than less typical objects, indicating a significant role of typicality in object recognition (Jolicoeur et al., 1984; Dell'acqua et al., 2000; Gastgeb et al., 2006). Related to within-category typicality, category agreement, measuring the degree of agreement among participants on the category the object belongs to, is another index reflecting how representative of its category an object is. Norms of category agreement were first introduced by Brodeur et al. (2010).

Third, the most recent image sets include a larger set of items that cover a wider range of categories. The original picture set in Snodgrass and Vanderwart (1980) included 260 item pictures that belonged to 14 categories. The new photo set introduced by Brodeur et al. (2010) includes 480 photo stimuli that belong to 18 different categories. Brodeur et al. (2010) recently extended their original photo set by adding 930 new normative photos (see also Duñabeitia et al., 2018, for a large set of colored pictures recently proposed). The newly included items and categories can better capture people's changing knowledge of object concepts, thus improving the ecological validity of the stimuli.

### 1.3. Motivations of the Present Study

Despite the various normative datasets of images, few original datasets are available with items that are selected to accommodate Chinese language and culture. We cannot emphasize enough on the importance of using a stimuli set tailored to a group's culture/language. First, and also obviously, participants from different culture and language backgrounds are expected to have variations in their norms. Thus, the norms obtained from one cultural/language group cannot be readily applied to another group with a different culture/language. This issue, however, can be relatively easily addressed by creating Chinese norms for the existing image sets. For instance, Chinese norms of the black and white line drawings from Snodgrass and Vanderwart (1980) were provided by Shu and Cheng (1989) and Zhang and Yang (2003). In addition, Weekes et al. (2007) provided the Chinese norms for the colored version of Snodgrass and Vanderwart (1980) based on Rossion and Pourtois (2004).

Second, and often easily to be ignored, some of the items that are easily recognizable in one culture/language might become unrecognizable in another. For instance, some of the fruits and vegetables that are so common in English-speaking countries, such as avocados and artichokes, are unfamiliar and even unknown to many of Chinese people. On the other hand, the Hami melon, a common fruit in China, might be unknown to some of the western counterparts. Then there are some of the unique objects that are only known to people who are familiar with Chinese culture, e.g., many of the music instruments such as the erhu and guzheng. Cross-cultural variation in the norms for the Snodgrass and Vanderwart (1980) image set was demonstrated in the study of Yoon et al. (2004), which reported a clear difference in the mean ratings of name agreement, concept agreement and familiarity between Chinese and American participants. Specifically, name agreement obtained from the Chinese group was significantly lower than that from the American group (see also Shu and Cheng, 1989). This problem is not unique to Chinese culture. Nishimoto et al. (2005) renormalized the Snodgrass and Vanderwart (1980) picture set for Japanese and found that their Japanese participants were not able to recognize some of the objects depicted in the original set. This issue cannot be addressed easily unless we create a new set of images that are carefully selected to accommodate the specific culture of the group.

However, most of the image sets available with Chinese norms are based on the line drawings from Snodgrass and Vanderwart (1980) or on some other datasets originally proposed for the English-speaking population decades ago. Recently, Zhou and Chen (2017) proposed a new set of 435 colored photos with Chinese norms. The new normative dataset is a big improvement over the previous Chinese normative datasets (Shu and Cheng, 1989; Zhang and Yang, 2003; Weekes et al., 2007; Liu et al., 2011), as it offers a set of colored photos rather than black and white or colored line drawings, thus improving the ecological validity of the dataset. However, all the item concepts in their stimuli set were obtained from Liu et al. (2011), of which the majority were collected from Cycowicz et al. (1997) (the remaining items being from various other English normative datasets). Therefore, the image set reflects the fact that the authors did not take the cross-cultural difference into account when selecting item concepts.

In the present study, we aimed to provide a new set of high-quality colored photos depicting a large number of items that are carefully selected to accommodate for Chinese culture. The new set of 551 photos, which covers the most categories so far, is normalized on 12 variables. Among the 12 variables, 10 of them were already introduced in previous studies, including name agreement (Snodgrass and Vanderwart, 1980; Alario and Ferrand, 1999; Bonin et al., 2003; Viggiano et al., 2004; Nishimoto et al., 2005), category agreement (Brodeur et al., 2010, 2014), familiarity (Snodgrass and Vanderwart, 1980; Alario and Ferrand, 1999; Bonin et al., 2003; Viggiano et al., 2004; Nishimoto et al., 2005; Brodeur et al., 2010), visual complexity (Snodgrass and Vanderwart, 1980; Alario and Ferrand, 1999; Bonin et al., 2003; Viggiano et al., 2004; Nishimoto et al., 2005; Brodeur et al., 2010), object manipulability (Magnié et al., 2003; Brodeur et al., 2010, 2014; Salmon et al., 2010), color diagnosticity (Rossion and Pourtois, 2004; Adlington et al., 2009), image variability (Snodgrass and Vanderwart, 1980; Alario and Ferrand, 1999; Bonin et al., 2003), AoA (Alario and Ferrand, 1999; Bonin et al., 2003; Nishimoto et al., 2005; Adlington et al., 2009), image agreement (Snodgrass and Vanderwart, 1980; Alario and Ferrand, 1999; Bonin et al., 2003; Brodeur et al., 2014) and within-category typicality (Dell'acqua et al., 2000; Lotto et al., 2001; Moreno-Martínez et al., 2011). The role of each of these 10 variables in object recognition and language processing has been discussed in the literature listed above. The remaining two variables, namely, shape diagnosticity and manipulation experience are introduced here for the first time.

#### 1.3.1. Shape Diagnosticity

The effect of global shape on object recognition has been tested in previous studies (Hayward, 1998; Lloyd-Jones and Luckhurst, 2002). Using the priming paradigm, Hayward (1998) found that recognition of an object can be facilitated by a priming stimulus that shares a similar outline shape. The influence of outline shape on object recognition, however, varies for living and non-living things. Outline shape benefits the recognition of living objects more than nonliving objects (Lloyd-Jones and Luckhurst, 2002). Given the important effect of outline shape on object recognition, we proposed to include shape diagnosticity as a new variable in our image set. Similar to color diagnosticity, we define shape diagnosticity as the degree to which an item is associated with a specific shape. If an item is strongly associated with a specific shape, i.e., the item has a unique shape, the shape information alone can inform us about its identity. On the other hand, if the item is weakly associated with any shape, i.e., it takes various forms in real life, we are unlikely to be able to recognize it based on its shape information alone.

#### 1.3.2. Manipulation Experience

Object manipulability is defined as the extent to which we can use our hands to grasp or use the item. It has been considered a good predictor of object recognition and thus was included as a variable in some of the previous image sets (e.g., Magnié et al., 2003; Brodeur et al., 2010). For instance, by using the priming paradigm, researchers have found that action representation associated with manipulating an object can be automatically activated upon viewing the object, which in turn facilitates recognition of a subsequent object that affords the same action (Ni et al., 2014; Helbig et al., 2006, 2010). The effect of manipulability on object recognition, however, is found to be mediated by the amount of manipulation experience associated with that object. The more experience we have in manipulating the object, the heavier role manual action plays in object representation (Yee et al., 2013; Chrysikou et al., 2017). Thus, we decided to include manipulation experience as another new variable in our image set. We define manipulation experience as how usual or often it is that people manipulate the item with their hands in their real life.

#### 1.3.3. Determinants of Naming Latency

An extensive number of studies have been conducted to reveal the determinants of picture naming latency. By using the line drawings taken from Snodgrass and Vanderwart (1980) and some other sources (e.g., Lotto et al., 2001; Bonin et al., 2003), the most consistently revealed predictors of picture naming latency include name agreement (Bonin et al., 2002, 2013; Alario et al., 2004; Alvarez and Cavanagh, 2004; Nishimoto et al., 2005, 2012; Weekes et al., 2007; Liu et al., 2011), image agreement (Bonin et al., 2002, 2013; Alario et al., 2004; Liu et al., 2011; Nishimoto et al., 2012), and AoA (Dell'acqua et al., 2000; Bonin et al., 2002, 2013; Alario et al., 2004; Alvarez and Cavanagh, 2004; Weekes et al., 2007; Liu et al., 2011; Nishimoto et al., 2012). Familiarity and image variability are reported as significant contributors to naming latency in some studies (familiarity: Snodgrass and Yuditsky, 1996; Ellis and Morrison, 1998; Weekes et al., 2007; Liu et al., 2011; image variability: Ellis and Morrison, 1998; Bonin et al., 2002; Alario et al., 2004) but not others (familiarity: Dell'acqua et al., 2000; Alario et al., 2004; Nishimoto et al., 2012; image variability: Liu et al., 2011; Nishimoto et al., 2012; Bonin et al., 2013). Variables such as visual complexity and within-category typicality are less robust predictors and found significant only in a few studies (Ellis and Morrison, 1998; Dell'acqua et al., 2000; Alario et al., 2004). More recently, object manipulability also has been reported as a significant predictor of naming latency (Guérard et al., 2015; Lorenzoni et al., 2018). The effects of category agreement, manipulation experience, and shape diagnosticity on picture naming latency have not been examined previously. Given the large number of variables rated in our photo set, we are in a better position to evaluate the determinants of naming latency.

## 2. General Method

### 2.1. Participants

We recruited 30 university students to rate each variable. Thus, to have all 12 variables rated, we recruited 360 participants in total (184 females, 176 males; average age 21.3, range: 18–28 years; years of education, range: 12–21 years). For the speed naming task, a new group of 28 university students (12 females, 16 males; average age 22.0; years of education: range 13–17 years) who did not participate in any of the rating tasks was recruited. All participants were native Mandarin Chinese speakers and had normal or corrected to normal vision. All participants provided consent forms before the normative session and received monetary rewards after the normative session. The study was approved by the Institutional Review Board of the Institute of Psychology, Chinese Academy of Sciences.

### 2.2. Stimuli

#### 2.2.1. Pre-selection

We first collected a large set of 824 original photos, enabling us to select among them the most suitable for Chinese culture. Apart from a small set of photos that was taken from the published photo set (Brodeur et al., 2010), the majority of the image set was downloaded for free from online sources. The original photo set was selected with three criteria. First, to create high-quality colored photos, we chose images only with a resolution higher than 500 × 500 pixels. Second, we only chose photos depicting items that are clearly segmented from the background. Third, we only chose photos depicting items with typical viewing perspectives that are commonly seen in daily life. Items shown with unusual viewpoints that obscure recognition were not included.

To further standardize the photo set, we took three more steps. We first set the background color to white by removing any background textures and colors in Photoshop. We then reset the sizes of all photos to 500 × 500 pixels. Note that we rescaled the whole photo size without distorting the original height/width ratio of the items depicted. Lastly, for objects with a long axis, we oriented the long axis to 45° or 135° to fit their functional use; e.g., the handle of an ax is orientated to 45° (Snodgrass and Vanderwart, 1980).

The original set of 824 photos depicted 399 unique items that belong to 21 categories, including reptiles, mammals, birds, fish, aquatic animals, insects, fruits, vegetables, nuts, office supplies, clothes, flowers, furniture, domestic appliances, vehicles, musical instruments, tools, weapons, kitchen wares, sporting goods, and daily supplies.

#### 2.2.2. Screening for Norms

We asked a group of 30 participants to name the original set of 824 photos. Based on the measures of name agreement, we removed 152 photos from the original image set either because they were unnamed (unrecognized) by more than 20% of the participants, or they were named incorrectly, or they had extremely low scores on name agreement (i.e., their dominant names were given by less than 20% of participants). The criterion for screening pictures based on name agreement measures was consistent with previous studies (Snodgrass and Vanderwart, 1980). We further excluded another 121 photos depicting different exemplars of the same items, e.g., different photos of water taps. We removed the duplicate exemplars of the same item because they either had lower name agreement or shared the same viewpoint. In the latter case, we randomly chose one exemplar to be included. However, we decided to keep some duplicate exemplars of the same items when they satisfied one of the following four criteria: (1) the different exemplars of the same item have large variations in appearance (e.g., various types of taps); (2) the different exemplars of the same item afford different manipulations (e.g., a water tap may afford a “press” or a“turn” action); (3) the different exemplars actually represent the different sub-types of the same item, e.g., both a low-speed train and a bullet train were kept because they represent different sub-types of train; (4) the different exemplars have different but equally common viewpoints. We were left with 551 photos, 57 of which were from the stimuli set of Brodeur et al. (2010). Note that 529 of the 551 photo stimuli representing 399 unique items were rated on the other 11 variables. The remaining 22 object photos were not assigned any category by our participants and thus were not rated on the variables of category agreement and within-category typicality. All the colored photos can be found in Supplementary Materials (File S1).

### 2.3. Procedure

#### 2.3.1. Rating Task

All photos were presented and rated on CRT screens with a resolution of 1024 × 768 and a refresh rate of 100 Hz. To measure name agreement, we presented the photo stimuli sequentially at the screen center by using C++ script. A blank box was presented right below each photo stimulus. Participants were instructed to type in the box the very first name of each item depicted that came to mind. To measure category agreement, we presented the modal name of each item with E-Prime 2.0 software and asked participants to select among 21 categories the one they thought the object best belonged to. To measure all other variables, we presented the photos, using the E-Prime 2.0 software at the screen center with a range of numbers 1–7 for AoA rating and 1–5 for all other ratings right below each photo stimulus. Participants were instructed to rate the variable by clicking on one of the five or seven numbers. Instructions were displayed on the screen and also given orally. All photos were randomly presented to each participant in each rating task. A small set of 28 practice stimuli was given to each participant before each normative session was conducted. The practice stimuli were not included in the final photo set. We recruited 30 participants to rate each of the 12 variables, and every 6 of the 30 participants were run in a group. Participants rated each of the photos sequentially and were self-paced. In the following section, we will specify the procedure of measuring each of the 12 variables, and the procedure of the speed naming task.

##### 2.3.1.1. Name agreement

Participants were instructed to type the very first name that came to mind once the photo stimulus appeared on the screen. They were told that the name could consist of more than one word. They were instructed to type “bu zhidao” (DKO, “don't know”) if they did not have any knowledge of the item depicted in the photo, “bu zhidao mingzi” (“don't know the name”) if they knew the object but did not know its name and “xiang bu qilai” (“TOT,” tip-of-the-tongue) if they knew the object but were not able to recall its name at that moment. Participants typed their answers in Chinese. There was no time constraint on the task.

##### 2.3.1.2. Familiarity

Following the definition of Snodgrass and Vanderwart (1980), we defined familiarity as “the degree to which you come in contact with or think about the concept.” Our participants were instructed to rate the familiarity based on how frequent or usual it is that they encounter or think of the item in their daily life. They were allowed to click DKO if they did not know the object. A 5-point rating scale was used in which 1 indicated very unfamiliar and 5 indicated very familiar. To avoid participants limiting themselves to a partial range of the Likert scale, we encouraged them to employ the whole range of numbers to rate the variables.

##### 2.3.1.3. Visual complexity

Visual complexity was reflected by the “amount of visual texture or intricacy of lines” of each item depicted. We emphasized that it did not refer to the conceptual complexity of the item. Participants rated it on a 5-point scale in which 1 indicated visually very simple and 5 indicated visually very complex.

##### 2.3.1.4. Manipulability

Object manipulability was defined as “the degree to which you are able to grasp or functionally use the item with hands in your daily life” (Magnié et al., 2003). Participants rated the variable on a 5-point scale, in which 1 indicated the item is very hard to be manipulated (i.e., grasped or used) with hands and 5 indicated the item is very easy to be manipulated with hands.

##### 2.3.1.5. Manipulation experience

We instructed participants to rate their manipulation experience with the items depicted in the photos. Related to object manipulability, manipulation experience was defined as “how usual/often you grasp or use the object with your hands in your daily life.” It was rated on a 5-point scale in which 1 and 5 indicated that the object was rarely or very often grasped or used in their daily life, respectively.

##### 2.3.1.6. Color diagnosticity

Color diagnosticity was defined as “the degree of association between the color and the item depicted” (Adlington et al., 2009). Participants rated the variable on a 5-point scale where 1 indicated that the object was weakly associated with the color and 5 indicated that the object depicted was strongly associated with the particular color.

##### 2.3.1.7. Shape diagnosticity

Similar to color diagnosticity, shape diagnosticity was defined as “the degree of association between the particular shape and the item depicted.” It was rated on a 5-point scale, where 1 and 5 indicated very weak or strong association between the item and the outline shape, respectively.

##### 2.3.1.8. Image variability

Image variability of an item was defined as “the number of different images of the item that you can think of” (Snodgrass and Vanderwart, 1980). We encouraged participants to recall as many different images related to that object as possible. Image variability was rated on a 5-point scale, in which 1 indicated very low image variability related to the image (i.e., the item evokes few different images) and 5 indicated high image variability of the item (i.e., the item evokes many different images).

##### 2.3.1.9. Age of acquisition (AoA)

AoA was defined as “the age at which you learned the name of the item depicted in the photo.” It was understandable that participants might not have a clear memory of when they began to learn each of the concepts. We provided them with seven age intervals of 0-2, 3-4, 5-6, 7-8, 9-10, 11-12, and >12 (Salmon et al., 2010) and asked them to choose one of them that indicated their AoA of the item name as accurately as possible.

##### 2.3.1.10. Image agreement

Participants were instructed to rate image agreement associated with the items depicted in the photos. Image agreement was “the degree to which your mental image of the item matches the one depicted in the photo” (Snodgrass and Vanderwart, 1980). Participants rated the variable on a 5-point scale in which 1 indicated the item depicted matched their mental image very badly and 5 indicated their mental image was almost the same as the item depicted.

##### 2.3.1.11. Category agreement

Category agreement was “the degree to which the object fits a specific category” (Brodeur et al., 2010). To measure this variable, we presented the modal name of each of the items along with 21 category names. Participants were asked to judge which category the item belonged to. Modal names provided for the items were based on the name agreement measures. Participants were allowed to select DKO (I don't know) if they had no idea which category the object belongs to. If participants thought the item belongs to none of the categories listed, they were allowed to click on “other” to indicate that the item belonged to a category not included.

##### 2.3.1.12. Typicality

Within-category typicality was defined as “the degree to which you think the item can represent the category it belongs to” (Moreno-Martínez et al., 2011). For example, how representative is “car” for the superordinate level category “vehicle?” Participants rated typicality on a 5-point scale where 1 and 5 indicated the item is a very poor or good exemplar of its category, respectively.

##### 2.3.1.13. Speed naming task

The procedure of the speed naming task was adopted from Bonin et al. (2002). A trial started with a fixation cross for 500 ms, followed by a photo stimulus presented at the screen center. The participants were required to speak out the name of the item depicted as accurately and quickly as possible. The photo stimulus was replaced by a blank screen immediately after the voice key was triggered. The blank screen remained for another 500 ms before the next trial began. The participants were allowed to say “I don't know” whenever they had no idea what the item depicted was and “I cannot remember” whenever they were not able to recall the item's name from their memory immediately. Participants were told to refrain from making meaningless sounds (e.g., “um” and “en”) that might trigger the onset of the voice recording. Reaction times were recorded via voice key. The experimenter sitting next to the participant monitored the participant's responses by recording any false triggers of the voice key, and any naming errors with a button box that was connected to the computer. Participants sat in a dim room in front of a CRT monitor (refresh rate: 100 Hz; resolution: 1024 × 768) with a viewing distance of approximately 70 cm. The procedure was programmed with the E-Prime 2.0 software.

The 399 unique object photos were randomly divided into 4 running blocks. After each block, the participants were given a 3-min break to ease their fatigue. Before the experimental session, participants practiced naming 20 photos (not included in the image set) to familiarize themselves with the trial procedure.

## 3. Data Analysis

### 3.1. Rating Task

As mentioned above, name agreement (NA) is defined as the degree to which all participants agree on the name of each item depicted. In line with the literature, we calculated two measures of name agreement. One is the percentage (%) of participants who give the same name for each item depicted and another is the *H* index, calculated by

$$H=\overset{k}{\underset{i=1}{\sum }}p_{i}\log_{2}\frac{1}{p_{i}}$$

where *k* indicates the number of different names all participants provided to each item and *p* is the proportion of participants providing each of the unique names for that item. The calculation of *H* did not include “don't know,” “don't know the name,” and “TOT” responses (see Snodgrass and Vanderwart, 1980). The higher the *H*, the lower the name agreement. *H* is considered to be a more sensitive measure of name agreement because it captures the wide distribution of all the names provided for each item (Snodgrass and Vanderwart, 1980).

Similarly, we calculated *H* and % values for category agreement. In this case, *k* indicates the number of unique category names all participants assigned to each item and *p* is the proportion of participants who agreed on each of the unique category names. Note that we calculated *H* and % values of category agreement based on the correct category name the participants agreed upon, even when the incorrect category name was agreed upon by a higher proportion of our participants. For instance, 10 out of 30 participants assigned “duck” to the wrong category “*mammal,”* and 9 participants assigned it to the correct category “*bird.”* We calculated *H* and % values only based on the rating score of the correct category “*bird.”* In addition, *H* and % values were not available for 22 items that were assigned by the majority of our participants to the unspecified category “*other.”*

Common to all variables, we calculated the mean, standard deviation, median, mode, minimum value, maximum value, 1st and 3rd quartiles, and skewness of the ratings. We also conducted correlation analysis among the 12 variables. To avoid overweighting the items with multiple exemplars, we chose to conduct statistical analysis only on the 399 unique items. For items with multiple exemplars, we selected the one with the highest name agreement. Thus, in the next section, we will only present the results based on the 399 unique objects.

#### 3.1.1. Speed Naming Task

To calculate the mean naming latencies, we first removed the trials in which the participants responded “I don't know” or “I cannot remember” (3.06%), the trials in which the participants named the items incorrectly (10.9%), and the trials in which the voice key of the microphone was triggered by unwanted sounds (e.g., “um” and “en”) (2.26%). We used the same criteria for defining correct responses as in Snodgrass and Yuditsky (1996). Specifically, a naming response was considered correct if (1) it was the dominant name of the item, as obtained from the name agreement session; (2) it was the abridged or more specific version of the dominant name (e.g., computer instead of personal computer or bullet train instead of train); (3) it was the main part of the dominant name. For example, although the dominant Chinese name for television is 电视机, literally translating to TV machine, it is also very common for the Chinese to say 电视(leaving out the “machine" part) . For each participant's data, the trials with naming times more than 2.5 standard deviations from the mean were also removed from further analysis (4.08 %) (see Liu et al., 2011).

## 4. Results and Discussion

### 4.1. Reliability of the Ratings

We first examined the inter-rater reliability for each of the 10 variables (excluding the variables of name agreement and category agreement, which were not suitable for the analysis) by calculating intraclass correlations (ICCs) via a two-way random consistency model (see Koo and Li, 2016, for correct model selection). ICCs revealed excellent reliability for familiarity (ICC = 0.94 [0.93, 0.95]), manipulability (ICC = 0.96 [0.97, 0.98]), manipulation experience (ICC = 0.97 [0.96, 0.98]), image variability (ICC = 0.96 [0.95, 0.97]), category typicality (ICC = 0.92 [0.90, 0.93]), AoA (ICC = 0.91 [0.88, 0.92]) and color diagnosticity (ICC = 0.97 [0.96, 0.98]); good reliability for visual complexity (ICC = 0.88 [0.86, 0.90]) and image agreement (ICC = 0.88 [0.86, 0.90]); and moderate reliability for shape diagnosticity (ICC = 0.72 [0.68, 0.76]).

### 4.2. Descriptive Statistics

**Figure 1:**
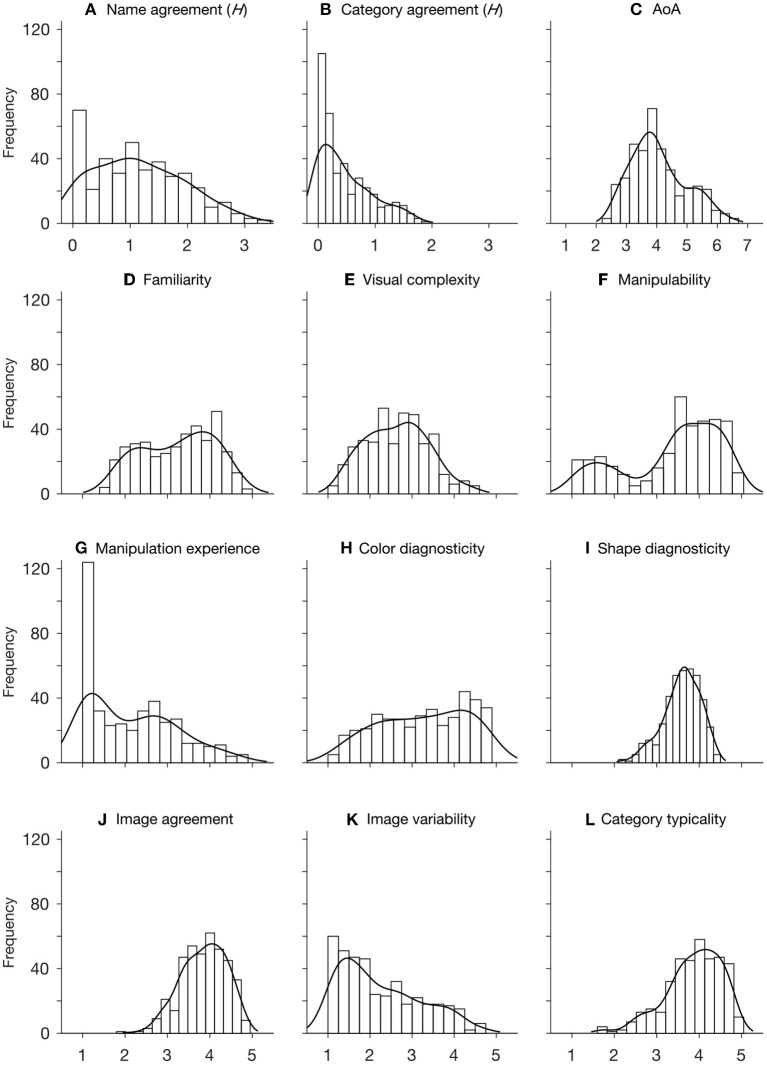
Histogram and distribution fit of each of the 12 variables. Histogram is fitted with nonparametric kernel-smoothing distribution. In **(A,B)**, numbers on *x*-axis represent the range of *H* value. In **(C)**, 1 to 7 correspond to the age intervals of 0-2, 3-4, 5-6, 7-8, 9-10, 11-12, and above 12 years old, respectively. In **(D–L)**, numbers on *x*-axis indicate the likert-scale of 5 points.

**Figure 2:**
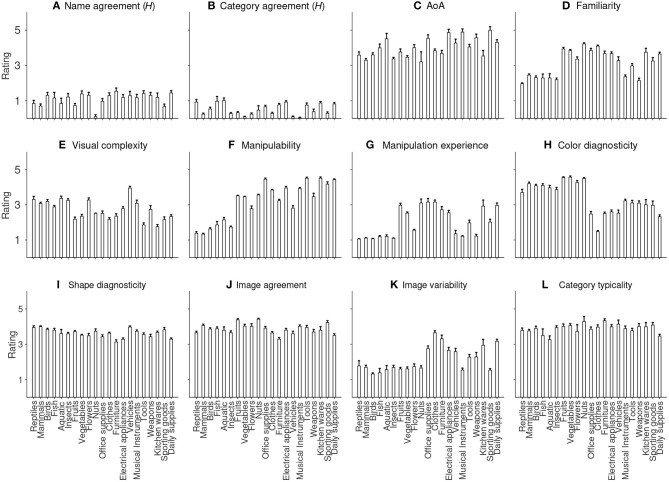
Average rating on each of the 21 categories for **(A)** Name agreement, **(B)** Category agreement, **(C)** AoA, **(D)** Familiarity, **(E)** Visual complexity, **(F)** Manipulability, **(G)** Manipulation experience, **(H)** Color diagnosticity, **(I)** Shape diagnosticity, **(J)** Image agreement, **(K)** Image variability, and **(L)** Category typicality. Error bars indicate one standard error above the mean rating.

We performed descriptive statistics on the ratings of all variables. As shown in Table 1, the distribution of *H* value has a mean of 1.16(±0.79), and a positive skewness, indicating a general high name agreement for the items. The high name agreement is also reflected by a mean % value of 0.69(±0.22) and a negative skewness. This is also true for category agreement, which has an even lower mean *H* value (0.48±0.47) and a higher % value (0.84±0.18). Together with the positive skewness of *H* distribution and negative skewness of % distribution, the ratings indicate that our participants reached very high agreements on which category each item should belong to (Figure 1B). The distribution of familiarity is approximately symmetric around the middle-point scale (Figure 1D), and the rating of visual complexity approximately follows a normal distribution (Figure 1E). The distribution of object manipulability has a mean (3.38±1.12) well above the middle-point. Together with a highly negative skewness (−0.71), it reflects that the majority of items could be manipulated to some degree (Figure 1F). Note that we also have a good number of items that were rated low on manipulability. Those objects are mostly living things such as animals and plants (see Figure 2F). In contrast, the mean rating of manipulation experience (2.16±1.03) is smaller than the middle point of the scale, and is highly positively skewed, indicating that our participants had relatively little experience in manipulating the items even though the items could be manipulated to various degrees. This is clearly demonstrated by the frequency distribution of manipulation experience, with its mode centering on 1 (see Figure 1G).

**Table 1:** Summary statistics for all variables.

	**NA**											**CA**		
	**H**	**%**	**Fam**	**VC**	**Manip**	**ME**	**CD**	**SD**	**IA**	**IV**	**AoA**	**H**	**%**	**CT**
Mean	1.16	0.69	3.24	2.63	3.38	2.16	3.26	3.59	3.86	2.25	4.06	0.48	0.84	3.88
Std	0.79	0.22	0.85	0.74	1.12	1.03	1.05	0.44	0.53	0.96	0.91	0.47	0.18	0.66
Median	1.06	0.73	3.34	2.67	3.63	2.00	3.35	3.63	3.92	1.97	3.90	0.35	0.92	4.00
Mode	0.00	1.00	4.09	1.87	3.43	1.00	4.13	4.00	4.00	1.53	3.33	0.00	1.00	4.00
Min	0.00	0.17	1.59	1.13	1.00	1.00	1.16	2.10	1.93	1.00	2.37	0.00	0.07	1.60
Max	3.44	1.00	4.88	4.57	4.93	4.85	4.90	4.47	4.93	4.69	6.63	1.94	1.00	4.93
Q1	0.56	0.50	2.44	2.07	2.64	1.15	2.39	3.33	3.50	1.39	3.38	0.00	0.77	3.53
Q3	1.75	0.87	4.00	3.13	4.27	2.88	4.19	3.93	4.27	2.92	4.59	0.77	0.97	4.37
Skewness	0.39	−0.35	−0.17	0.14	−0.71	0.58	−0.22	−0.58	−0.39	0.65	0.54	0.90	−1.32	−0.79

*NA, name agreement; Fam, familiarity; VC, visual complexity; Mainp, manipulability; ME, manipulation experience; CD, color diagnosticity; SD, shape diagnosticity; IA, image agreement; IV, image variability; AoA, age of acquisition; CA, category agreement; CT, within-category typicality; Std, standard deviation; Q1, 1st quartile; Q3, 3rd quartile; Skewness, Pearson's moment coefficient of skewness*.

The distribution of color diagnosticity is moderately negatively skewed with a mean (3.26±1.05) and a mode (4.13) above the middle-point of the scale, reflecting that the majority of the items we selected have moderate to high color diagnosticity. The distribution of shape diagnosticity has a high mean (3.59±0.44) and mode (4.00). Together with a highly negative skewness, this indicates that the majority of the items are strongly associated with their particular shapes (Figure 1I). The distribution of image agreement has a mean (3.86±0.53) that is well above the middle-point of the scale, and is highly negatively skewed, indicating that most of the items depicted match fairly well with our participants' mental images.

In contrast, the majority of the items have a lower-than-middle-point score on image variability, which is also reflected by the highly positive skewness of the rating distribution (Figure 1K). The Q1 and Q3 of AoA distribution are 3.38 and 4.59, respectively, indicating that our participants learned 50% of the item names roughly between the ages of 5 and 9 years old. The average rating of within-category typicality is 3.88±0.66, which is well above the middle point of the scale. Together with the highly positive skewness, this indicates that most of the items depicted are good exemplars of their respective categories.

**Figure 3:**
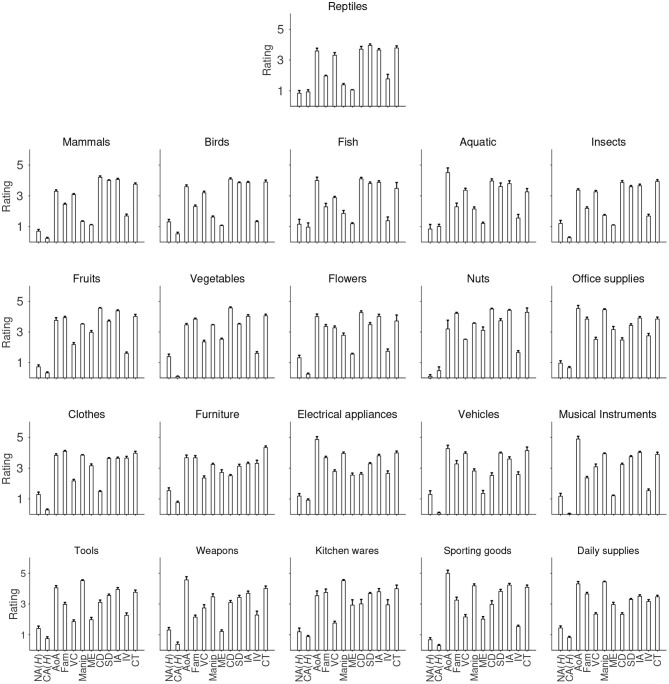
Average rating of each of the 12 variables across the 21 categories. NA *H*, Name agreement; CA*H*, category agreement; AoA, Age of acquisition; Fam, familiarity; vC, visual complexity; Manip, manipulability; ME, manipulation experience; CD, color diagnosticity; SD, shape diagnosticity; IM, image agreement; IV, image variability; CT, within-category typicality. Error bars indicate one standard error above the mean.

Figure 2 shows the mean ratings of each of the 21 categories on each variable. While there is no evident difference in the mean ratings for most of the variables, interesting patterns can be seen for a few of them. Object manipulability, as pointed out above, is relatively lower for living objects, such as reptiles, mammals, birds and fish (Figure 2F). That is also true for manipulation experience (Figure 2G). Living things also tend to be rated lower on familiarity compared to non-living things such as tools (Figure 2D). By contrast, non-living things in general have lower scores on color diagnosticity than living things (Figure 2H). Moreover, most of the non-living categories, such as clothes, furniture and domestic appliances, have higher image variabilities than living categories (Figure 2K). The average rating for each category across the 12 variables is clearly displayed in Figure 3.

### 4.3. Correlations Among Variables

Apart from the descriptive statistics, we also conducted Pearson correlations among all the variables (see Table 2). Note that the 0.05 and 0.01 significance levels were Bonferroni corrected for multiple correlations.

**Table 2:** Pairwise correlations between the 12 Variables.

	**Variables**	**1**	**2**	**3**	**4**	**5**	**6**	**7**	**8**	**9**	**10**	**11**	**12**	**13**
1	Name agreement (*H*)	–	–	–	–	–	–	–	–	–	–	–	–	–
2	Name agreement (%)	−0.94^**^	–	–	–	–	–	–	–	–	–	–	–	–
3	Famliarity	−0.10	0.18^*^	–	–	–	–	–	–	–	–	–	–	–
4	Visual complexity	−0.02	−0.00	−0.42^**^	–	–	–	–	–	–	–	–	–	–
5	Manipulation	0.09	−0.04	0.58^**^	−0.52^**^	–	–	–	–	–	–	–	–	–
6	Manipulation experience	−0.05	0.11	0.88^**^	−0.51^**^	0.64^**^	–	–	–	–	–	–	–	–
7	Color diagnosticity	−0.11	0.06	−0.26^**^	0.16	−0.50^**^	−0.36^**^	–	–	–	–	–	–	–
8	Shape diagnosticity	−0.33^**^	0.34^**^	0.00	0.22^**^	−0.28^**^	−0.13	0.30^**^	–	–	–	–	–	–
9	Image agreement	−0.39^**^	0.35^**^	0.19^**^	−0.11	−0.02	0.07	0.42^**^	0.48^**^	–	–	–	–	
10	Image variability	0.04	0.05	0.50^**^	−0.26^**^	0.45^**^	0.56^**^	−0.67^**^	−0.24^**^	−0.40^**^	–	–	–	–
11	Age of acquisition	0.25^**^	−0.26^**^	−0.13	0.14	0.27^**^	−0.08	−0.22^**^	−0.28^**^	−0.09	−0.12	–	–	–
12	Category agreement *(H)*	0.04	−0.04	0.08	−0.15	0.19^*^	0.17	−0.18^*^	−0.17	−0.13	0.24^**^	0.12	–	–
13	Category agreement (%)	−0.07	0.09	−0.06	0.14	−0.24^**^	−0.16	0.20^**^	0.21^**^	0.15	−0.24^**^	−0.16	−0.91^**^	–
14	Category typicality	−0.04	0.11	0.33^**^	0.00	0.03	0.25^**^	0.02	0.28^**^	0.18^*^	0.09	−0.17	−0.32^**^	0.47^**^

* p < 0.05;

** *p < 0.01*.

Not surprisingly, we found a strong negative correlation between the two measures (*H* and %) of name agreement (−0.94), which is consistent with previous studies (e.g., Brodeur et al., 2010; Zhou and Chen, 2017). The same is true for the correlation between *H* and % of category agreement (−0.91). The negative correlation of −0.42 between familiarity and visual complexity is quite comparable to −0.47 reported by Snodgrass and Vanderwart (1980) (see Shu and Cheng, 1989; Weekes et al., 2007; Zhou and Chen, 2017, for similar results). The negative correlation could be accounted for by the observation that visually less complex objects tend to be non-living objects (e.g., man-made tools), which are more familiar to our participants (see Figures 2D,E). We also found a modest positive correlation between modal name agreement (*p* value) and familiarity (0.18), which is consistent with some of the previous results (Weekes et al., 2007; Brodeur et al., 2010; Liu et al., 2011; Zhou and Chen, 2017) but not others (Snodgrass and Vanderwart, 1980; Zhang and Yang, 2003). For instance, whereas Snodgrass and Vanderwart (1980) and Zhang and Yang (2003) found no significant correlation between name agreement and familiarity, Adlington et al. (2009) reported a significant correlation as high as 0.71. Importantly, we found a decent positive correlation between object manipulability and familiarity (0.58) and a negative correlation between object manipulability and visual complexity (−0.52), both of which are higher than reported in the preceding studies (Magnié et al., 2003; Brodeur et al., 2010; Salmon et al., 2010).

One of our new findings is that manipulation experience related to an object strongly positively correlates with familiarity (0.88), which is not surprising given that the more experience we have in manipulating the items (e.g., tools), the more familiar we are with them. Manipulation experience is also negatively correlated with visual complexity (−0.51), probably due to the fact that easily manipulated objects tend to be visually simple, small objects such as tools (see Figure 2G). As would be expected, manipulation experience is positively correlated with the degree of object manipulability (0.64).

Color diagnosticity is weakly and negatively correlated with familiarity (−0.26) as compared to no significant correlation reported by Adlington et al. (2009). Our explanation for the weak correlation is that visually more complex items are usually living things (e.g., animals) that are less familiar to us (see Figures 2D,E). More surprisingly, color diagnosticity has a decent negative correlation with object manipulability (−0.50) and a modest negative correlation with manipulation experience (−0.36). We interpreted the negative correlations as being associated with the fact that in our photo set, items with high color diagnosticity are usually from categories such as animals, which have low ratings on both object manipulability and manipulation experience (see Figures 2E–G).

We are the first to report a modest positive correlation between shape diagnosticity and name agreement (−0.33 and 0.34 for *H* and % values, respectively), indicating that the higher the name agreement, the higher the shape diagnosticity. Shape diagnosticity also weakly correlates with visual complexity in a positive direction (0.22), probably due to the fact that visually complex items (e.g., animals) are not as likely to take different forms/shapes in life as visually simple objects like man-made small tools. Shape diagnosticity also has a negative correlation with object manipulability (−0.28). The negative correlation would be explained by the fact that the items with unique shapes associated with them tend to be living objects that could not be easily manipulated. Shape diagnosticity also modestly and positively correlates with color diagnosticity (0.30), suggesting that objects with unique colors (i.e., most of which are living things) also tend to have unique shapes associated with them.

Image agreement has a positive correlation of 0.35 with name agreement (% value), quite comparable to those reported previously (Snodgrass and Vanderwart, 1980, 0.31; Zhang and Yang, 2003, 0.38; Brodeur et al., 2010, 0.33; Liu et al., 2011, 0.39; Zhou and Chen, 2017, 0.39). We also found a small but significant positive correlation between image agreement and familiarity (0.19), which is slightly higher than reported by Snodgrass and Vanderwart (1980) (0.14), Shu and Cheng (1989) (0.15), and Weekes et al. (2007) (0.13) but lower than reported by Zhang and Yang (2003) (0.44), Brodeur et al. (2010) (0.38), Liu et al. (2011) (0.42), and Zhou and Chen (2017) (0.26). Image agreement also has modest positive correlations with both color diagnosticity (0.42) and shape diagnosticity (0.48), which could be understood in the way that objects with specific colors and shapes tend to have less variability in mental images.

Image variability correlates positively with familiarity (0.50), which is not surprising given that we usually have more different mental images of items that we are highly familiar with. However, the correlation is stronger than reported in the normative datasets of Bonin et al. (2003) (0.20) and Liu et al. (2011) (0.19). Image variability also has a negative correlation with visual complexity (−0.26), consistent with that reported by Bonin et al. (2003) (−0.24). The negative correlation is not hard to interpret as visually complex items (e.g., animals) tend to have limited variations in forms/shapes (see Figures 2E,K). Thus, people's mental images of them also tend to be fixed. Image variability positively correlates with object manipulability (0.45) and manipulation experience (0.56). These suggest that frequently manipulating an object helps us build different mental images of that object. Image variability is negatively correlated with color diagnosticity (−0.67) and shape diagnosticity (−0.24), suggesting that items that lack specific colors and shapes associated with them usually have more different mental images. As expected, we found a negative correlation between image variability and image agreement (−0.40), which is quite comparable to the those reported by Snodgrass and Vanderwart (1980) (−0.44) and Bonin et al. (2002) (−0.36). The negative correlation is expected simply because we can hardly reach a high agreement on the mental image of an object if it is associated with many different mental images.

AoA is weakly negatively correlated with name agreement (0.25 and −0.26 for *H* and % values, respectively), which is comparable to the results reported in some of the previous studies (e.g., Bonin et al., 2003; Liu et al., 2011; Zhou and Chen, 2017), but not others. For example, Adlington et al. (2009) reported a negative correlation as high as −0.82, but Snodgrass and Vanderwart (1980) and Weekes et al. (2007) observed a non-significant correlation between the two. Our results suggest that the earlier you learned the concept of an item, the more likely you associate with it a specific name. We did not observe a significant correlation between AoA and familiarity, which is unexpected as a strong negative correlation between the two variables was observed in most of the previous datasets (Snodgrass and Vanderwart, 1980; Snodgrass and Yuditsky, 1996; Cycowicz et al., 1997; Bonin et al., 2003; Weekes et al., 2007; Adlington et al., 2009; Liu et al., 2011; Moreno-Martínez et al., 2011; Ghasisin et al., 2015). AoA is also weakly positively correlated with object manipulability (0.27), suggesting that the objects that could be easily manipulated by our hands (e.g., man-made tools) tend to be learned at a later age. To our best knowledge, the only other study that has both AoA and object manipulability rated is Moreno-Martínez et al. (2011), which reported however a positive but non-significant correlation (0.19) between the two. Importantly, AoA is also found negatively correlated with both color diagnosticity (−0.22) and shape diagnosticity (−0.28), indicating that it is easier to learn the names of items that are strongly associated with particular colors or global shapes. The only study that examined the correlation between AoA and color diagnosticity reported a non-significant correlation between the two (Adlington et al., 2009).

We observed a weak negative correlation between category agreement and object manipulability. Given the small magnitude in correlation (0.19 for *H* and −0.24 for %), we are not surprised that the correlation was absent in Brodeur et al. (2010). Category agreement has a weak positive correlation with color diagnosticity (0.20 for % value) and shape diagnosticity (0.21 for % value), suggesting that the items with more variations in either color or shape are harder to categorize. Category agreement also has a negative correlation with image variability (−0.24), which is expected as image variability and image agreement are negatively correlated.

We found that within-category typicality is positively correlated with familiarity (0.33), which is comparable to the correlation of 0.46 reported by Dell'acqua et al. (2000), but is lower than the 0.92 reported by Moreno-Martínez et al. (2011). The positive correlation with familiarity is expected as familiar items are usually good exemplars of the category they belong to. Typicality also positively correlates with manipulation experience (0.25), which might be mediated by familiarity, as more manipulation experience usually leads to higher familiarity. In addition, we are the first to observe that within-category typicality correlates positively with shape diagnosticity (0.28) and image agreement (0.18), reflecting that objects associated with few shapes, and thus having higher image agreement usually are good exemplars of their category. Typicality is also positively correlated with category agreement (−0.32 and 0.47 for *H* and % values, respectively), suggesting that people have higher category agreement for the objects that are considered to be good exemplars of their category.

It should be noted that the pairwise correlations between the 12 variables are relatively small except for a few pairs that are expected to be moderate or high (e.g., high correlation between *H* and % values of name agreement and category agreement, and moderate correlation between object manipulability and manipulation experience). Small magnitudes in correlations suggest that our variables represent independent attributes of the stimuli (see also Snodgrass and Vanderwart, 1980).

The correlation matrix obtained from the CIS is largely consistent with those reported in the literature. The only substantial difference is the absence of a significant correlation between AoA and familiarity that is commonly observed in the preceding normative datasets (Snodgrass and Vanderwart, 1980; Bonin et al., 2003; Adlington et al., 2009; Moreno-Martínez et al., 2011; Ghasisin et al., 2015). A closer look at the data reveals that most of the object names were learned by our participants before the age of 10, as indicated by a higher positive skewness than those observed in (all, as far as we know) previous studies (Snodgrass and Vanderwart, 1980; Cycowicz et al., 1997; Alario and Ferrand, 1999; Bonin et al., 2003; Liu et al., 2011; Zhou and Chen, 2017). The fact that few items were learned at a later age could attenuate its correlation with familiarity whose distribution follows a somewhat bimodal distribution (see Figure 1). We believe that the high positive skewness of AoA distribution is a result of delibarate efforts to select a stimuli set that accommodates Chinese language and Culture. In contrast, all the previous datasets, including those providing Chinese norms (Liu et al., 2011; Zhou and Chen, 2017) consist of object items that were originally chosen within English-speaking culture, resulting in some of the objects being unfamiliar or even barely known to the Chinese population.

### 4.4. Multiple Regression on Naming Latency

The average naming latency across all items was 1,216 ms, which is quite comparable to the results for Chinese speakers reported by Bates et al. (2003) (1,200 ms), Weekes et al. (2007) (1,025 ms), Liu et al. (2011) (1,044 ms), Zhang and Yang (2003) (1,354 ms), and Zhou and Chen (2017) (1,039 ms). However, it is worth noting that the reported naming latency in Chinese was longer than those reported in other languages, such as English (Snodgrass and Yuditsky, 1996, 791 ms; Ellis and Morrison, 1998, 794 ms), Spanish (Cuetos et al., 1999, 829 ms), and Turkish (Bakhtiar et al., 2013, 916 ms). The difference might be the result of a large number of polysyllabic words (e.g., compound words consisting of multiple syllables) in Mandarin Chinese, as suggested by Bates et al. (2003).

#### 4.4.1. Stepwise Multiple Regression Analysis

To evaluate the contribution of each of the 12 variables to naming latency, we conducted a stepwise multiple regression including the variables in Table 2. Note that for name agreement and category agreement, only the *H* values were entered into the regression analysis as they were highly correlated with their corresponding % values (see also Weekes et al., 2007; Nishimoto et al., 2012).

As displayed in Table 3, the significant predictors were name agreement (*H* value), AoA, shape diagnosticity, image variability, and image agreement. Specifically, the items that scored high on name agreement and image agreement and were learned at an early age were named faster than those that were scored low on them and were learned at a later age. The results are in agreement with the previous studies indicating that name agreement, AoA, and image agreement are the three most frequently reported predictors of naming latency (e.g., Barry et al., 1997; Bonin et al., 2002, 2013; Alario et al., 2004; Liu et al., 2011; Nishimoto et al., 2012; Zhou and Chen, 2017). In addition, image variability is also a reliable determinant of naming latency reported here, consistent with some of the preceding studies showing that items with high image variability were named faster than those with low variability (Ellis and Morrison, 1998; Bonin et al., 2002; Alario et al., 2004). Moreover, it is of interest that shape diagnosticity, which is newly introduced in our dataset, is also shown as a robust predictor of naming latencies. Specifically, the items with highly diagnostic shapes tend to be named faster than those with low diagnostic shapes. The result highlights the role of shape information in object recognition (see also Hayward, 1998; Lloyd-Jones and Luckhurst, 2002). Together, the five variables accounted for 51.8% (adjusted *R*^2^) of the total variance [*F*_(5, 393)_ = 86.64, *p* < 0.001]. None of the other variables in Table 2 contributed significantly to the naming latency.

**Table 3:** Stepwise multiple regression on naming latencies.

**Predictors**	**β Coeff**.	**Standard error**	**t value**
Name agreement(*H*)	0.29	0.039	7.52^**^
Age of acquisition	0.20	0.037	5.35^**^
Shape diagnosticity	−0.26	0.041	−6.16^**^
Image variability	−0.38	0.039	−9.78^**^
Image agreement	−0.26	0.045	−5.69^**^

** *p <0.01*.

## 5. General Discussion

Image stimuli have been extensively used to probe various cognitive processes, including object recognition, memory, and language processing. Amongst the different stimuli sets that have been proposed, the standardized picture set introduced by Snodgrass and Vanderwart (1980) is arguably the most well-known and widely used stimuli set. Although its contribution to the research community has been enormous and indisputable, its limitations have emerged over the years. First, the picture set consists of 260 line drawings that are short on surface details, such as color and texture. Such surface details have been shown to be important in object representation. Second, it provides a relatively small set of object pictures, some of which may not be as common as they used to be 30 years ago. For instance, by comparing with the original measures from Snodgrass and Vanderwart (1980), Yoon et al. (2004) found that the measures in name agreement and concept agreement percentages had changed for a good number of the images over the decades. These two points together highlight the issue that the original picture set from Snodgrass and Vanderwart (1980) has been losing its ecological validity over the years. Although Rossion and Pourtois (2004) added texture and color to the original picture set from Snodgrass and Vanderwart (1980), the images are still of poor quality as compared to the colored photos. Third, in the original picture set, only five variables—name agreement, image agreement, familiarity, visual complexity and image variability—were normalized. Though these five variables have proven to be important psycholinguistic factors, new variables such as manipulability and color diagnosticity also have been shown to modulate the memory and cognitive processing. Thus, it entails the inclusion of new variables into the later proposed image sets.

Norms of the picture set from Snodgrass and Vanderwart (1980) have been provided for a wide range of other languages, including Chinese (Shu and Cheng, 1989; Zhang and Yang, 2003; Weekes et al., 2007; Liu et al., 2011). While Chinese norms for the picture set provide useful tools for examining various cognitive processes within the Chinese culture, they inherit some of the limitations from Snodgrass and Vanderwart (1980). Specifically, the black and white line drawings with Chinese norms still lack in surface details and cover only a limited range of object categories. Moreover, some of the pictures in Snodgrass and Vanderwart (1980) are less recognizable in Chinese culture. This is evidenced by the fact that name agreement for the Snodgrass and Vanderwart (1980) picture set was lower amongst Chinese speakers than English speakers (Shu and Cheng, 1989; Zhang and Yang, 2003; Weekes et al., 2007; Liu et al., 2011). To overcome these issues, new datasets of high-quality colored images have been proposed recently to improve the ecological validity (Adlington et al., 2009; Brodeur et al., 2010, 2014; Moreno-Martínez et al., 2011). Among them, Zhou and Chen (2017) proposed the first set of colored photos with Chinese norms. However, as mentioned earlier in the introduction, Zhou and Chen (2017) did not take Chinese culture and language into consideration when selecting the items. Moreover, some of the variables (e.g., manipulability, color diagnosticity, within-category typicality) that recently have been shown to be important factors in language and object processes are not included in their normative dataset.

The present study provides a new dataset of photo stimuli that are carefully selected for Chinese speakers. The new normative photo set (CIS) covers more than 21 object categories and is rated on 12 variables, amongst of which manipulation experience and shape diagnosticity are introduced and normalized for the first time. Our new dataset enjoys good inter-rater reliability as revealed by the intraclass correlations. The high validity of our dataset is also corroborated by the correlation analysis among the 12 variables. Specifically, the magnitudes of pairwise correlations between the variables are within reasonable ranges, suggesting that the variables measured in the study have good construct validity. The correlations we observed are largely consistent with those reported in the previous studies with one exception: we did not find a significant negative correlation between AoA and familiarity. A negative correlation between those two variables is expected because intuitively the earlier we learn an object concept, the more familiar we would be with it. The possible reason for the absence of a significant correlation, as discussed in the result section, is that the distribution of AoA is highly positively skewed, indicating that few items are learned at a later age by our participants, whereas the distribution of familiarity resembles a bimodal distribution. The highly skewed AoA distribution, as we have argued earlier on, results from the fact that the objects' concepts were selected to reflect Chinese language and culture. Consequently, unlike previous stimulus sets, very few object items were deemed unrecognizable in our dataset.

Multiple regression analysis on naming latency reveals the five most robust predictors, including name agreement, image agreement, AoA, image variability, and shape diagnosticity. Amongst them, name agreement, image agreement, and AoA are the most robust and consistently reported determinants of naming latency in the literature. Image variability is also reported as a significant predictor in previous studies, though less consistently. Note that we are the first to measure the variable of shape diagnosticity. Although it is not unexpected to see it contributing significantly to naming latency as its role in object recognition has been demonstrated previously (Hayward, 1998; Lloyd-Jones and Luckhurst, 2002), we should consider this variable with caution because the inter-rater reliability of shape diagnosticity is only modest. However, we would like to suggest that researchers in the future should include shape diagnosticity as a potentially important variable into their new datasets and confirm its role in naming latency and object recognition in general.

Contrary to our expectations, object manipulability did not contribute to naming latencies. Prior studies examining its role in object recognition have consistently found that manipulation information is heavily involved in object processing (e.g., Helbig et al., 2006, 2010; Ni et al., 2014, 2019). For instance, compared to low-graspability objects, high-graspability objects were named faster (Guérard et al., 2015; Lorenzoni et al., 2018). Our explanation for the null result is that the effect of manipulation on object processing is mediated by manipulation experience, as evidence has indicated that the causal role of action information in objects' conceptual representations is established through manual experience (Yee et al., 2013; Chrysikou et al., 2017). The distribution statistics of manipulation experience, nevertheless, show that our participants had little experience in acting upon most of the objects in our dataset in spite of their high scores on manipulability.

Our new photo set was normalized amongst young college students, with their ages ranging from 18 to 28 years old. Obtaining norms in a young population, especially amongst college students is not an uncommon practice in the literature (e.g., Snodgrass and Vanderwart, 1980; Shu and Cheng, 1989; Zhang and Yang, 2003; Nishimoto et al., 2005; Weekes et al., 2007). Although this does not preclude the norms from being widely used because the majority of the studies testing cognitive processes recruited young participants, it gives rise to the issue of generalizability of our dataset to other age groups. Yoon et al. (2004) compared normative measures on the picture set from Snodgrass and Vanderwart (1980) between younger and older adults within both Chinese and American cultural groups, and reported higher scores on name agreement and concept agreement for a small subset of data among younger Americans, compared with older American adults. In contrast, another small subset of pictures yielded lower name agreement and concept agreement for older compared with younger adults. Moreover, although the correlations in measures between younger and older American adults were significantly high for the majority of the pictures (i.e., around 80% of the entire picture set), they become non-significant for the remaining pictures with low name and concept agreement percentages. This is largely true within the Chinese cultural group as well. The age-related difference also manifests itself when comparing norms between children and adults. Evidence has indicated that the measures of name agreement, familiarity, and visual complexity from the children group were lower than those from the adults group (Pompéia et al., 2001, see also Berman et al., 1989; Cycowicz et al., 1997, for the difference between children and adults). Within Chinese culture in particular, Wang et al. (2014) found a significant difference in familiarity rating between preschool children and adults. Preschool children tended to rate familiarity lower than adults did. The age-related difference in measures was also demonstrated in other datasets (Sirois et al., 2006). Given the weight of the evidence, we have good reasons to be conservative with the generalizability of the dataset to younger and older populations. Future work should be devoted to select part of the photo set suitable for larger populations.

In sum, by standardizing a new set of high-quality colored stimulus photos, we offer an ecologically more valid tool to study object recognition and language processing within the context of Chinese language and culture.

## Data Availability Statement

All datasets generated for this study are included in the article/Supplementary Material.

## Ethics Statement

The study was approved by the Institutional Review Board of the Institute of Psychology, Chinese Academy of Sciences.

## Author Contributions

LN, YL, WY, and XF conceived and designed the study, and wrote the paper. LN collected and analyzed the data.

### Conflict of Interest

The authors declare that the research was conducted in the absence of any commercial or financial relationships that could be construed as a potential conflict of interest.

## Supplementary Material

The complete stimulus set along with the norms can be found: https://www.dropbox.com/sh/zyafrk6gv02qk42/AADu43y78qPwNXOW3d5kvJLga?dl=0.

File S1 A total number of 552 photos, including 399 unique items (File 1) and 152 duplicate items (File 2).

Data Sheet (Excel) Norms for the complete set of 552 items (sheet 1), the subset of 399 unique items (sheet 2), and the subset of 152 duplicate items (sheet 3).
